# Case Report: Multiple Cavernous Pericardial Lymphangioma (Pericardial Lymphangiomatosis) in a Captive Peregrine Falcon (*Falco peregrinus brookei*)

**DOI:** 10.3389/fvets.2021.662157

**Published:** 2021-04-14

**Authors:** José Espinosa, M. Carmen Ferreras, David García, Raquel Vallejo, Valentín Pérez

**Affiliations:** ^1^Departamento de Sanidad Animal, Facultad de Veterinaria, Instituto de Ganadería de Montaña (CSIC-ULE), Universidad de León, León, Spain; ^2^Falco Iberia S.L., León, Spain

**Keywords:** *Falco peregrinus*, lymph vessels, lymphangiomatosis, lymphangioma, pericardium

## Abstract

A 12-year-old female peregrine falcon (*Falco peregrinus brookei*) from a private raptor breeding facility that presented a good body condition, died suddenly without showing previous symptoms. At necropsy, in the coelomic cavity, multiple cystic structures demarcated by a thin transparent wall and filled with a serous content were observed. They were firmly adhered to the cranial part of the epicardium and adjacent tissues and occupied the entire thoracic area of the coelomic cavity. Microscopically, emerging simultaneously from several areas the epicardium, multiple irregular channels and cystic spaces, lined by a single endothelial cell layer and separated by fibrovascular septa containing smooth muscle tissue, were observed. Immunohistochemical examination revealed that the neoplastic endothelial cells positively immunolabelled for the pan-endothelial marker factor VIII-related antigen but immunostained negative for cytokeratins (PCK26) while strong positivity for sarcomeric α-smooth muscle actin (α-SMA) was detected in the cystic walls. Based on the morphological and immunohistochemical findings, lesions were determined as consistent with a multiple cavernous pericardial lymphangioma, or pericardial lymphangiomatosis, a rare vascular neoplasm. The animal also showed a diffuse chronic perihepatitis, a necrotic area in the liver and foci of cartilaginous metaplasia and calcification in the aorta and vena cava. Literature review, particularly on the epidemiology of lymphangioma, demonstrated the rarity of this tumor in the different animal species and in this location, particularly in birds, being the first report of this type of tumor in a peregrine falcon.

## Background

The growing interest in wildlife is behind the increase in the number of reports of different pathologies affecting these species, which is leading veterinary medicine to expand the range of clinicopathological knowledge in this area. However, at present, the available information is still very limited in comparison to domestic species. In the specific case of neoplastic conditions of wild birds, studies on their nature and frequency are scarce and little is known about their etiology, pathogenesis or biological behavior. In addition, the number of recent and updated studies is very limited and those available are mainly focused on companion or aviary birds (e.g., psittacine birds), with the result that incidence of neoplasia is higher in these avian species compared to the rest (1–6). In contrast, in birds of prey, tumors are considered as relatively rare. Worldwide, neoplasms have been reported in more than 40 species among the families Accipitridae, Falconidae, Strigidae and Cathartidae, and a wide variety of tumors have been described (7–12). Despite this, limited data are available in relation to the prevalence of neoplasia, species, sex or age predisposition. Tumor nomenclature is inconsistent and etiology, oncogenesis, and biological behavior are basically unknown. Furthermore, it is considered that the information included in those reports does not correspond to the real prevalence of neoplasia in these species due to the marked shortage of cases documented in the literature. In the recent decades, the number of birds of prey kept in long-term captivity, for breeding with conservation purposes or for falconry activity, has increased considerably. Greater longevity, inbreeding or exposure to environmental or nutritional carcinogens (13) may increase the risk of developing neoplasms, including types of tumors not registered so far. This fact will allow to improve the understanding and documentation of these pathological conditions in these species. In this line, this study describes the main pathological and immunohistochemical findings of a multifocal pericardial lymphangioma (pericardial lymphangiomatosis) in a 12-year-old captive peregrine falcon (*Falco peregrinus brookei*), a rare vasoformative pathology of neoplastic nature that, to the authors' knowledge, has not been reported to date in birds and is poorly documented in other animal species.

## Case History

A 12-year-old female captive peregrine falcon was referred on January 2020 to the Pathological Diagnostic Service of the Faculty of Veterinary Sciences of the University of León, Spain, to determine the possible cause of death. The bird was imprinted on its owner and was part of a captive breeding program since it was 2 years old. The animal died suddenly without showing obvious clinical signs, apart from a loss of vocalization just before death, and was immediately submitted for necropsy. The animal had shown reproductive failure after the last artificial insemination. Feeding was based on defrosted 1-day-old chicks and quails, without extra food supplementation. The animal showed a very good body condition at the time of death, with an *in vivo* bodyweight of 980 gr. Given the rapidity of the process, no previous hematological and biochemical analyses, neither other clinical tests, were performed.

## *Post-Mortem* Diagnosis

### Necropsy and Gross Findings

**Figure 1:**
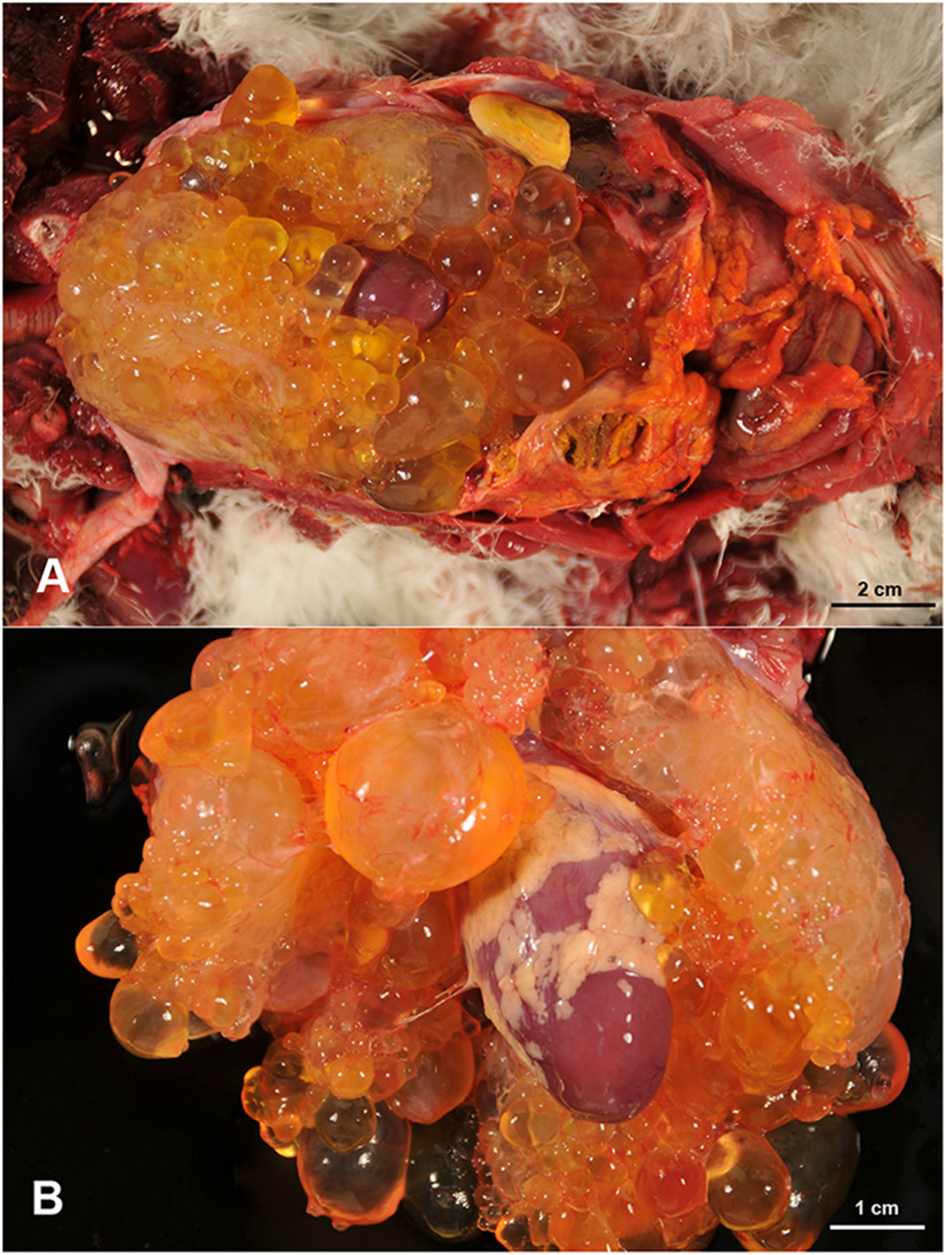
Multiple cystic cavities, filled with a clear fluid, are occupying the thoracic area of the coelomic cavity with presence of free fibrin clots **(A)**. These cystic structures engulfed the heart and are firmly attached to the epicardium **(B)**.

At necropsy, the presence of abundant subcutaneous fat and appropriate sizes of the skeletal muscles, which appeared normal, was noticed. In the coelomic cavity, abundant cloudy free sero-hemorrhagic content (50 mL), that coagulated easily on contact with air, as well as the presence of fibrin clots, was observed. The liver was enlarged and showed a thickened, rough capsule, firmly adhered to the parenchyma. A 2.5 cm in diameter area of greenish caseous necrotic material, surrounded by a thin fibrous capsule, was present focally adhered to the liver capsule. In the coelomic cavity, multiple cystic structures, ranging from 0.3 cm to 2 cm in diameter, demarcated by a thin wall but easily separated one from each other, and containing serous, clear and transparent content, were observed (Figure 1A). These cysts formed a single mass that was strongly adhered to the epicardium and adjacent structures, hindering its separation from the heart and related vessels (Figure 1B). The cystic mass extended up to the bifurcation of the trachea and occupied the entire thoracic area of the coelomic cavity. Among these cystic cavities, abundant yellowish-white strands of a slightly elastic material, consistent with fibrin, were noticed. The lungs were mildly congested and the heart appeared normal, with no alterations in the size of cardiac chambers or in the valves. No other gross lesions were detected in the rest of the organs. Representative tissue samples were collected from the lesions and the rest of the organs, for histopathological examination.

### Histopathological and Immunohistochemical Studies

Tissue samples fixed in 10% buffered formalin were routinely processed through a graded alcohol series and xylene, before being embedded in paraffin wax. Tissue sections 2.5 μm thick were obtained from each sample and stained with Harris's haematoxylin and eosin (H&E). Additional stains were performed, including Periodic acid-Schiff (PAS), Masson trichrome or Gram and Ziehl-Neelsen stains. Moreover, two direct smears, together with two others made from the liquid extracted from the caelomic cavities after centrifugation and sediment collection, were fixed in methanol, stained using the May–Grünwald Giemsa method and mounted for cytological examination.

To characterize the lesions, immunohistochemical tests were performed from sections of the cystic structures observed in the coelomic cavity. Antibodies against anti smooth muscle α-actin (α-SMA) (Monoclonal; Clone 1A4; 1:100, Dako-Agilent^®^ technologies, Santa Clara, USA), cytokeratins (Monoclonal; Clone PCK26; 1:100, Dako-Agilent^®^), factor VIII-related antigen (polyclonal; 1:100; Dako-Agilent^®^), and Prospero-related homeobox gene-1 (Prox-1) (Monoclonal; Clone 5G10; 1:100, Thermo Fisher Scientific^®^, Massachusetts, USA) were used. The manufacturers had reported, through a verified customer review, that the different antibodies showed cross-reactivity in sections of formalin-fixed avian tissues (chicken); however, they could not guarantee this reactivity in avian species others than chicken. Heat mediated antigen retrieval was performed by means of PT Link^®^ system, using the pH 9.0 and pH 6.0 target retrieval solutions (Dako-Agilent^®^) for 20 min at 96°C for α-SMA and factor VIII-related antigen, respectively, and a trypsin antigen retrieval solution (Abcam, Cambridge, UK) for 15 min at 37°C in the case of the cytokeratin antibody. For the Prox-1 antibody, different unmasking protocols, including the use of pH 6.0 and 9.0 antigen retrieval solutions and trypsin, as previously mentioned, were tested. After deparaffinization, rehydration and drying, sections were immersed into a 3% H_2_O_2_ in methanol solution for 30 min at room temperature and darkness to block endogenous peroxidase. Immunolabelling was performed using a ready-to-use kit EnVision System^®^ (Dako-Agilent^®^) where slides were incubated for 40 min at room temperature. After washing twice in PBS, antibody localization was determined using 3,3-diaminobenzidine (Dako-Agilent^®^) as chromogenic substrate for peroxidase. Finally, slides were counterstained with Harris haematoxylin. Appropriate species-and isotype-matched immunoglobulins were used as control. These included sections with an isotype control for the primary antibody, and the omission of the primary antibody. As positive controls, the same examined sections were used to determine the antibody optimal dilution and incubation pH and included several normal tissues from healthy chickens.

## Histopathological Findings

**Figure 2:**
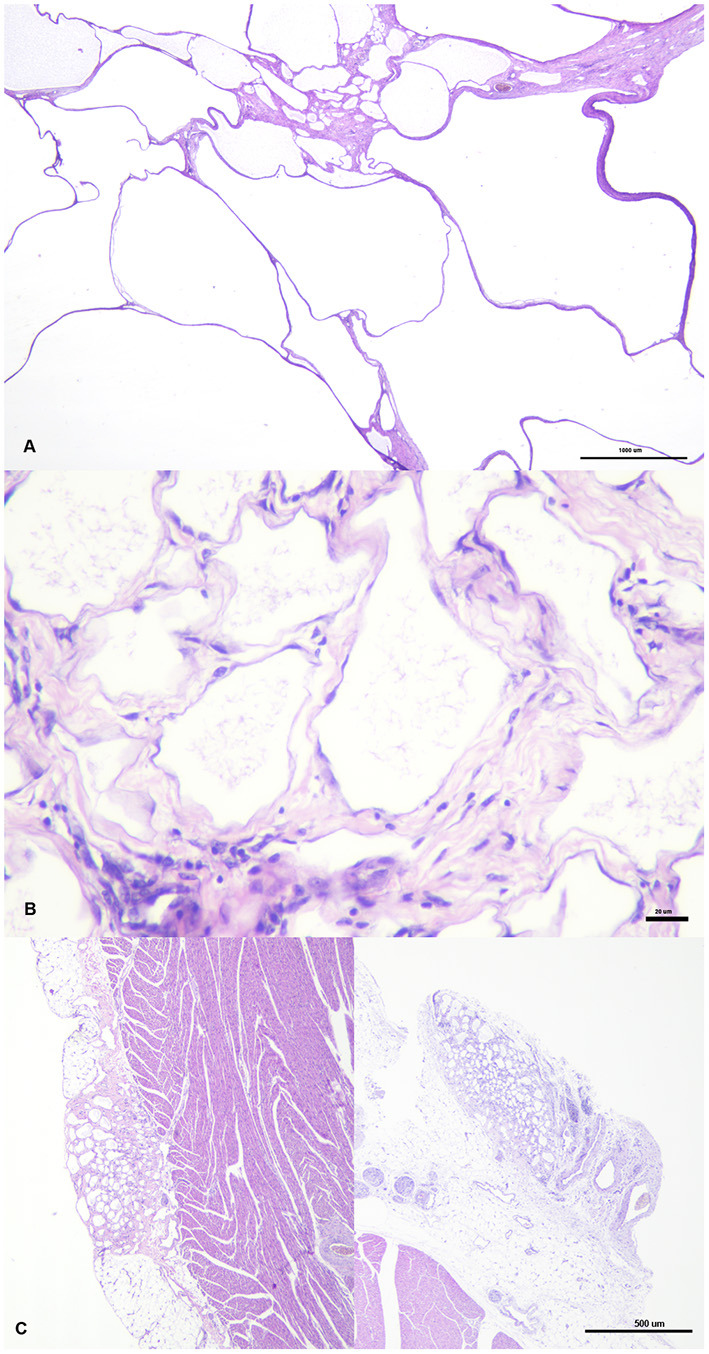
Cystic structures have different size and are separated by septa of variable thickness **(A)**. They are lined by endothelial cells surrounded by fibrovascular tissue, where fusiform cells and occasional aggregates of lymphocytes and plasma cells are seen **(B)**. Two foci or lymphatic vessel proliferation, arising from the epicardium, simultaneously at two different point can be seen on the left and right pictures **(C)**.

**Figure 3:**
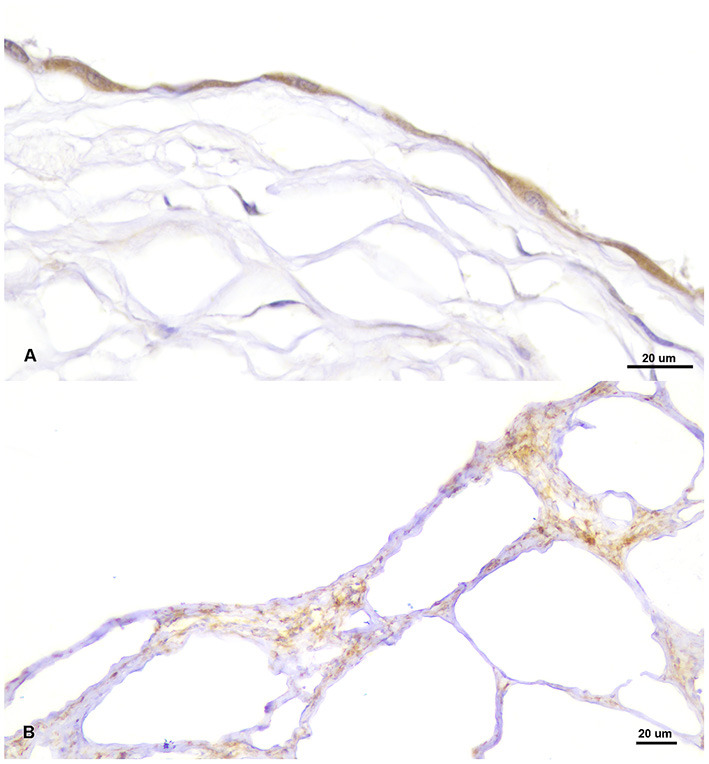
Positive immunolabelling for pan-endothelial marker factor VIII-related antigen in the endothelial cells lining the cystic cavities **(A)** and for smooth muscle actin (α-SMA) in the septum that separate the cysts **(B)**.

Sections of the cystic mass revealed the presence of multiple irregular cavernous or macrocystic spaces, consistent with multiple dilated lymphatic channels, separated by thin to moderately thick fibrovascular stromal septa, where the presence of fusiform cells separated by an eosinophilic matrix, blood vessels of different sizes, and a multifocal, mild, inflammatory infiltrate composed of lymphocytes, plasma cells and occasional foamy cells, were observed (Figure 2A). These spaces were lined by a single endothelial cell layer that showed mild pleomorphism, with a flattened cell shape, scarce basophilic homogenous cytoplasm and oval to triangular, centrally located, nuclei, mostly hyperchromatic (Figure 2B). These cavities were frequently filled with a pale, slightly eosinophilic fluid with the occasional presence of sloughed cells. All the cellular components were generally well-differentiated and lacked cellular atypia or the presence of mitotic figures. The origin of these cystic structures was determined to be the epicardium where, histologically, similar lymphatic vessels of more reduced size, were seen multifocally arising from this layer (Figure 2C). By PAS staining, the absence of a well-defined linear basal membrane was noticed and the presence of a subendothelial muscle layer was detected by Masson's trichrome staining. Immunohistochemical examination revealed that the neoplastic endothelial cells expressed the pan-endothelial marker factor VIII-related antigen (Figure 3A) but lacked immunoreactivity for cytokeratin, while a strong positive immunolabelling to α-SMA was seen in cells located in the septa, consistent with the presence of a layer of smooth muscle in the cystic wall (Figure 3B). Based on these findings, a final diagnosis of multiple cavernous pericardial lymphangioma, or disseminated pericardial lymphangiomatosis, was made. No immunolabelling for Prox-1 antibody was found in the endothelial cells that lined the cystic spaces, or in any of the lymphatic structures of the rest of the normal tissues of the affected bird, in all of the immunohistochemical trials performed. Meanwhile, sections of normal chicken tissue showed positive immunostaining against Prox-1 in cell lining structures consistent with lymph vessels.

The aortic artery and cranial vena cava showed focal areas of smooth muscle cell degeneration, with a vacuolated appearance, at the level of the tunica media, moderate disruption of elastic laminae, and the presence of calcification foci and areas of cartilaginous metaplasia. On the wall of some bronchi there were also areas of mineralization and cartilaginous metaplasia, as well as proliferation of myxoid tissue between the bronchial layers. Mineralized *corpora amylacea* were observed in some thyroid follicles. The caseous and greenish focus grossly observed in the liver was formed by an area of caseous necrosis affecting the liver parenchyma, surrounded by an inflammatory infiltrate composed of heterophils, foamy macrophages, foreign body type giant cells as well as cholesterol crystals. In addition, this organ showed a moderate diffuse perihepatitis where lymphocytes, plasma cells and heterophils were infiltrating the liver capsule, together with multifocal inflammatory foci of heterophils scattered in the liver parenchyma. Bacteria were not observed after Gram or Ziehl-Neelsen stains. No noticeable microscopic changes were detected in the rest of the organs.

The fluid extracted from the caelomic cavities was characterized cytologically by presenting low cellularity, good cellular preservation and the existence of a very low amount of blood. Only mesothelial cells in different stages of activation were observed, characterized by a central round nuclei and moderate bluish cytoplasm, or the existence of more abundant vacuolated cytoplasm and an eccentric nucleus, leading to the diagnosis of a modified transudate. Cell atypia or other cytological features consistent with neoplasia were not observed, neither the presence of bacteria in any of the smears evaluated.

## Review and Discussion

Tumors of lymphatic vessels are rare in humans and domestic animals but particularly in birds since, although they have lymphatic channels, these are less developed than those in mammals (14). After reviewing the available literature, only three cases of avian lymphangiomas have been reported, and none of them were similar in terms of their anatomical location and clinical features (2, 15, 16). Moreover, in the rest of animal species, we have not found references regarding lymphangiomas with an origin at the pericardial level (see Table 1).

**Table 1:** Number of cases of benign tumors of lymphatic vessels (lymphangiomas and lymphangiomatosis) in the different animal species, other than humans, reported in the literature.

**Animal species**	**Neoplasm type (cases)**	**Anatomical location (number of cases)**	**References**
Dog (*Canis lupus familiaris*)	Lymphangioma Cystic lymphangioma Multiple invasive lymphangioma	Cutaneous (2), nasopharynx (1), retroperitoneum (1), axilla (1), spleen (1), medial aspect of thigh (1), mammary region and lymph node (1), inguinolabial region (1)	(17–26)
	Cystic lymphangiomatosis	Liver (1), pelvic limb (1), cutaneous (1), abdominal cavity (1)	
Domestic cat (*Felis silvestris catus*)	Multiple cavernous lymphangioma	Liver (1), cutaneous (1)	(27, 28)
Budgerigar (*Melopsittacus undulatus*)	Lymphangioma	Speen (1)	(2)
Chimney Swift (*Chaetura pelagic*)	Lymphangioma	Skin (neck area) (1)	(15)
Darwin's Rhea (*Pterocnemia pennata*)	Lymphangioma	Mediastinum (1)	(16)
Sprawe-Dawley rat (*Rattus norvegicus*)	Lymphangioma	Skin (3), liver (1)	(29)
Horse (*Equus caballus*)	Cystic lymphangioma	Abdominal and retroperitoneal area (1), cutaneous (2), submandibular area (1)	(30–34)
	Cystic lymphangiomatosis	abdominal cavity (1)	
Jaguar (*Panthera onca*)	Lymphangioma	Omentum (1)	(7)
Squirrel monkey (*Saimiri sciureus*)	Cavernous lymphangioma	Kidney (1)	(35)
Leopard (*Panthera pardus*)	Lymphangioma	Mesentery (1)	(7)
Lamb (*Ovis aries*)	Bilateral lymphangioma	Testicle (1)	(36)
Mongolian gerbil (*Meriones unguiculatus*)	Lymphangioma	Liver (1)	(37)
Mount carmel blind mole-rat (*Spalax carmeli*)	Cystic lymphangiomatosis	Abdominal cavity (1)	(38)
Ongole bull (*Bos tauro*)	Cystic lymphangioma	Scrotal area (1)	(39)
Tequila fish (*Zoogoneticus tequila*)	Lymphangiomatosis	Spleen (1)	(40)
White-Tailed Deer (*Odocoileus virginianus*)	Cavernous lymphangioma	Liver (1)	(41)

Lymphangiomas, as an isolated lesion, are benign, slow-growing tumors of the lymphatic system and are regarded as malformations arising from sequestration of lymphatic tissue that fail to communicate with the lymphatic system (42, 43). Traditionally, lymphangiomas have been classified according to their histological appearance into capillary, cavernous, and cystic (44). In the present case, the lesion reported was characterized histologically by a cavernous appearance due to the presence of widely dilated lymphatic channels, with fibrovascular adventitial coats, as well as by its intrathoracic location inside of the coelomic cavity. On the other hand, the term lymphangiomatosis is used to describe the presence of multiple lymphangiomas affecting soft tissue or parenchymal organs in a diffuse or multifocal fashion (42, 45). The presence of multiple cavernous lymphangiomas, arising multifocally from different pericardial areas, was consistent with the condition reported in this case.

Basic knowledge that may help to explain the etiology of these lymphatic lesions is missing. In general, it is accepted that the main cause is a congenital failure of the lymphatic system to connect with, or separate from, the venous system, or an abnormal budding of lymphatic tissue from the cardinal vein (46, 47). However, in humans, it has been observed that there are stimuli that can trigger the proliferation of neoplastic lymphatic endothelial cells, such as infections, trauma, chronic inflammation or obstruction (48, 49). In birds, some types of vasoformative neoplasms arise as sequelae of some viral infections such as avian leukosis (11, 16). In the present case, the etiology of the pericardial lymphangiomatosis reported is unknown and we cannot conclude, from the pathological results, whether this is a congenital or an acquired process. An area of hepatic necrosis and the presence of inflammatory foci in the liver parenchyma were found, for which their exact causes could were not determined. However, it seems unlikely that this lesion has triggered the development of pericardial lymphangiomas due to its hepatic location and its subacute character. On the other hand, a congenital origin cannot be completely ruled out despite the adult age of the bird. Intrathoracic lymphangiomas may remain asymptomatic for many years and only became apparent when clinical signs derived from the compression of vital structures appear (40, 50). In this case, an acute heart failure secondary to the mechanical action of the large multicystic mass could be the main cause of death. Moreover, the large amount of free fluid into the coelomic cavities is consistent with hydrostatic disorders associated with a progressive heart failure.

On the other hand, degenerative changes characterized by the fragmentation and disruption of the elastic fibers, together with foci of dystrophic calcifications and cartilaginous metaplasia, were observed in the tunica media of the aortic artery and cranial vena cava. These changes have been associated with atherosclerosis, mainly in psittacine species and captive birds of prey (51, 52); however, in this case, no histological changes consistent with this condition were found. Other causes such as copper deficiency or hypertension have also been associated with these degenerative changes (53, 54). In this case, copper levels and the existence of alterations in blood flow were not evaluated. The mechanical effect of cystic masses over the great vessels of the heart that might have contributed to the appearance of these vessel wall lesions, could not be ruled out.

Lymphangiomas have to be distinguished from other vascular cystic lesions such as multicystic mesothelioma, haemangioendothelioma or lymphangiosarcoma. All of them are rare vasoformative tumors and are difficult to differentiate due to similarities in their macroscopic and histological findings; therefore, immunohistochemical studies are necessary for their differential diagnosis. As in this case, lymphangiomas are characterized by the presence of endothelial cells with immunoreactivity for endothelial marker factor VIII-related antigen and lack cytokeratin expression, particularly cytokeratin 5 and 6 (recognized by the antibody employed), together with the lack of a clearly identified basal lamina and the existence of a layer of smooth muscle in the cystic wall, as demonstrated by the positive immunolabelling for α-SMA. These findings can be used to differentiate lymphangioma from multicystic mesotheliomas or haemangioendotheliomas (55–57). Prox-1 marker was tested since it is reported to be expressed by lymphatic endothelium (58, 59). However, baseline studies in birds are very scarce (58), with no previous results in raptors. In our case, no positive immunolabelling was observed in any of the lymphatic tissues of the affected bird, although it was detected in chicken tissues used as positive controls, reaching the conclusion that the marker used does not show immunoreactivity in the peregrine falcon tissues. Thus, all the histological and immunohistochemical findings support the diagnosis of lymphangioma. This tumor and lymphangiosarcoma only differ in their degree of cellular pleomorphism and mitotic activity. In the present case, the neoplastic cells were mildly pleomorphic and mitotic figures were not observed, features highly consistent with a lymphangioma.

In conclusion, this appears to be the first report of a multiple cavernous pericardial lymphangioma or pericardial lymphangiomatosis in a peregrine falcon. This case is of additional importance due to the scarcity of reports of this condition at the heart level, not only in birds, but also in other mammals including man. At present, few cases of neoplasms in birds of prey continue to be reported, so that every effort should be made to send suspected samples originating from captive o free-living birds for pathological investigation and, subsequently, improve the understanding of the prevalence of neoplastic diseases in the different avian species.

## Data Availability Statement

The original contributions generated for this study are included in the article/Supplementary Material, further inquiries can be directed to the corresponding author/s.

## Ethics Statement

Ethical review and approval was not required for the animal study because the procedures were performed on an animal on the demand of the owner (Falco Iberia S. L.). The study was carried out on the basis of the clinical situation, required by the owner of the animal. Written informed consent was obtained from the owners for the participation of their animals in this study.

## Author Contributions

JE and VP contributed to the writing of the manuscript and literature review. JE, MF, RV, and VP contributed to the necropsy and histopathological examination, interpretation and description of the histopathological and immunohistochemical results. DG was responsible for the follow-up of the case and the submission of the animal to necropsy. All authors discussed the results and commented on the manuscript.

## Conflict of Interest

The authors declare that the research was conducted in the absence of any commercial or financial relationships that could be construed as a potential conflict of interest.

## References

[1] Frost C. Experiences with pet budgerigars. Vet Rec. (1961) 73:621–26.

[2] Petrak ML Gilmore CE. Neoplasms. In: Petrak ML editor. Diseases of Cage and Aviary Birds. 2nd ed. Philadelphia: Lea & Febiger London: Bailiiere. Tindall & Cassell (1982). p. 606–37.

[3] Beach JE. Diseases of budgerigars and other cage birds. A survey of postmortem findings. Vet Rec. (1962) 74:63–8.

[4] Blackmore DK. The clinical approach to tumours in cage birds—I: the pathology and incidence of neoplasia in cage birds. J Small Anim Pract. (1966) 7:217–23. 10.1111/j.1748-5827.1966.tb04435.x 5949541

[5] Reavill DR. Tumors of pet birds. Vet Clin Exot Anim. (2004) 7:537–60. 10.1016/j.cvex.2004.04.008 15296864

[6] Castro PF Fantoni DT Miranda BC Matera JM. Prevalence of neoplastic diseases in pet birds referred for surgical procedures. Vet Med Int. (2016) 2016:1–7. 10.1155/2016/4096801 26981315 PMC4770162

[7] Ratcliffe HL. Incidence and nature of tumors in captive wild mammals and birds. Am J Cancer Res. (1933) 17:116–35. 10.1158/ajc.1933.116577508

[8] Keymer IF. Diseases of birds of prey. Vet Rec. (1972) 90:579–94. 10.1136/vr.90.21.579 5073130

[9] Reece RL. Observations on naturally occurring neoplasms in birds in the state of Victoria, Australia. Avian Pathol. (1992) 21:3–32. 10.1080/03079459208418815 18670912

[10] Effron M Griner L Benirschke K. Nature and rate of neoplasia found in captive wild mammals, birds, and reptiles at necropsy. J Natl Cancer Inst. (1977) 59:185–98. 10.1093/jnci/59.1.185 577508

[11] Forbes NS Cooper JE Higgins RJ. Neoplasms in birds of prey. In: Lumeij JT Remple JD Redig PT Lierz M Cooper JE editors. Raptor Biomedicine III. Lake Worth, Florida: Zoological Education Network (2000). p. 127–46.

[12] Cooper JE. Birds of Prey: Health and Disease. 3rd ed. Oxford: Blackwell Science (2008). p. 200–1.

[13] Latimer KS. Oncology. In: Ritchie BW Harrison GJ Harrison LR editors. Avian Medicine: Principles and Application. Lake Worth, Florida: Wingers Publishing (1994). p. 640–72.

[14] Wilting J Aref Y Huang R Tomarev SI Schweigerer L Christ B. Dual origin of avian lymphatics. Dev Biol. (2006) 292:165–73. 10.1016/j.ydbio.2005.12.043 16457798

[15] Dickinson JC. Chimney swift having benign lymphangioma. Auk. (1941) 58:581–81. 10.2307/4078662

[16] Hubbard GB Schmidt RE Fletcher KC. Neoplasia in zoo animals. J Zoo Anim Med. (1983) 14:33–40. 10.2307/20094627

[17] Post K Clark EG. Gent IB. Cutaneous lymphangioma in a young dog. Can Vet J. (1991) 32:747–8. 17423917 PMC1481132

[18] Stambaugh JE Harvey CE Goldschmidt MH. Lymphangioma in four dogs. J Am Vet Med Assoc. (1978) 173:759–61 711598

[19] Yamagami T Takemura N Washizu T Komori S Amasaki H Washizu T. Hepatic lymphangiomatosis in a young dog. J Vet Med Sci. (2002) 64:743–45. 10.1292/jvms.64.743 12237525

[20] Belanger MC Mikaelian I Girard C Daminet S. Invasive multiple lymphangiomas in a young dog. J Am Anim Hosp Assoc. (1999) 35:507–9. 10.5326/15473317-35-6-507 10580911

[21] Berry WL Nesbit JW Pearson J. Lymphangiomatosis of the pelvic limb in a Maltese dog. J Small Anim Pract. (1996) 37:340–43. 10.1111/j.1748-5827.1996.tb02405.x 8840256

[22] Woods JP Johnstone IB Bienzle D Balson G Gartley CJ. Concurrent lymphangioma, immune-mediated thrombocytopenia, and von Willebrand's disease in a dog. J Am Anim Hosp Assoc. (1995) 31:70–6. 10.5326/15473317-31-1-70 7820768

[23] Maeda S Fujino Y Tamamoto C Suzuki S Fujita A Takahashi M. Lymphangiomatosis of the systemic skin in an old dog. J Vet Med Sci. (2013) 75:187–90. 10.1292/jvms.12-0321 22986273

[24] Oui H Lamm C Stiver S William B Know SY Bae Y. Congenital lymphangiomatosis and an enteric duplication cyst in a young dog. J Small Anim Pract. (2014) 55:379–82. 10.1111/jsap.12208 24628429

[25] Ramírez GA Sánchez-Salguero X Molín J. Primary cystic lymphangioma of the spleen in an adult dog. J Comp Pathol. (2020) 178:22–26. 10.1016/j.jcpa.2020.06.006 32800104

[26] Sato Y. Cutaneous pedunculated lymphangioma in a dog. J Small Anim Pract. (2021). 10.1111/jsap.13301. [Epub ahead of print]. 33522616

[27] Lawler DF Evans RH. Multiple hepatic cavernous lymphangioma in an aged male cat. J Comp Pathol. (1993) 109:83–7. 10.1016/S0021-9975(08)80242-3 8408783

[28] Santos S Faísca P. Feline cutaneous lymphangioma: case report. RECIL. (2012) 5:73–6. Available online at: https://revistas.ulusofona.pt/index.php/rlcmv/article/view/3021

[29] Mackenzie WI Gardner FM. Comparison of neoplasms in six sources of rats. J Natl Cancer Inst. (1973) 50:1345. 10.1093/jnci/50.5.1243 4712589

[30] Turk JR Gallina AM Liu IM. Cystic lymphangioma in a colt. J Am Vet Med Assoc. (1979) 174:1228–30. 438053

[31] Gehlen H Wohlsein P. Cutaneous lymphangioma in a young Standardbred mare. Equine Vet J. (2000) 32:86–8. 10.2746/042516400777612017 10661392

[32] Junginger J Rötting A Staszyk C Kramer K Hewicker-Trautwein M. Identification of equine cutaneous lymphangioma by application of a lymphatic endothelial cell marker. J Comp Pathol. (2010) 143:57–60. 10.1016/j.jcpa.2009.11.005 20042195

[33] Hoepp NC Kim DY Berent LM Reed SK. What is your diagnosis? Fluid surrounding a submandibular mass from a horse. Vet Clin Pathol. (2013) 42:531–32. 10.1111/vcp.12078 24102533

[34] Savage VL Cudmore LA Russell CM Railton DI Begg AP Collins NM. Intra-abdominal cystic lymphangiomatosis in a Thoroughbred foal. Equine Vet Educ. (2018) 30:403–8. 10.1111/eve.12685

[35] King CS Streett JW Brownstein DG. Cavernous lymphangioma in a squirrel monkey. Lab. Anim. Sci. (1993) 43:252–54. 8355487

[36] Brown P Smith K Bazely K Glover M Barr F. Bilateral lymphangiomatous testicular lesions in a lamb. Reprod Domest Anim. (2008) 43:246–48. 10.1111/j.1439-0531.2006.00841.x 18226019

[37] Vincent AL Porter DD Ash LR. Spontaneous lesions and parasites of the Mongolian gerbil, *Meriones unguiculatus*. Lab Anim Sci. (1975) 25:711–22. 1207042

[38] Sós E Molnár V Gál J Németh A Perge E Lajos Z. Typhlitis and abdominal cystic lymphangiomatosis in a Mt. Carmel blind mole rat (*Nannospalax (ehrenbergi) carmeli*). J Zoo Wildl Med. (2012) 43:416–20. 10.1638/2011-0201.1 22779253

[39] Kumar RS Veena P Devarathnam J Amaravati P Sudarshan LS. Surgical management of a rare case of scrotal lymphangioma in ongole bull. J Adv Vet Res. (2014) 4:85–7.

[40] Romanucci M Arbuatti A Defourny SVP Della Salda L. Multiple, life-compatible, congenital physical deformities in association with splenic lymphangiomatosis in *Zoogoneticus tequila* (Webb & Miller, 1998). J Coast Life Med. (2017) 5:1–3. 10.12980/jclm.5.2017J6-280

[41] Chute HL Chamberlain DM. Lymphangioma in the white-tailed deer, *Odocoileus virginianus*. J Mammal. (1956) 37:552–54. 10.2307/1376664

[42] Faul JL Berry GJ Colby TV Ruoss SJ Walter MB Rosen GD. Thoracic lymphangiomas, lymphangiectasis, lymphangiomatosis, and lymphatic dysplasia syndrome. Am J Respir Crit Care Med. (2000) 161:1037–46. 10.1164/ajrccm.161.3.9904056 10712360

[43] Blei F. Lymphangiomatosis: clinical overview. Lymphat Res Biol. (2011) 9:185–90. 10.1089/lrb.2011.0020 22196283

[44] Mentzel T Kutzner H. Tumors of the lymphatic vessel of the skin and soft tissue. Der Pathologe. (2002) 23:118–27. 10.1007/s00292-001-0498-9 12001527

[45] Weiss SW Goldblum JR Enzinger FM. Tumors of lymph vessels. In: Weiss SW Goldblum JR editors. Enzinger and Weiss's Soft Tissue Tumors. 4th ed. Philadelphia, PA, USA: Elsevier Health Sciences (2001). p. 966–9.

[46] Perkins JA Manning SC Tempero RM Cunningham MJ Edmonds JL Jr. Hoffer FA. Lymphatic malformations: current cellular and clinical investigations. Otolaryngol Head Neck Surg. (2010) 142:789–94. 10.1016/j.otohns.2010.02.025 20493347

[47] Zheng W Aspelund A Alitalo K. Lymphangiogenic factors, mechanisms, and applications. J Clin Invest. (2014) 124:878–87. 10.1172/JCI71603 24590272 PMC3934166

[48] Wiegand S Eivazi B Barth PJ Von Rautenfeld DB Folz BJ Mandic R. Pathogenesis of lymphangiomas. Virchows Arch. (2008). 453:1–8. 10.1007/s00428-008-0611-z 18500536

[49] Rockson SG. Causes and consequences of lymphatic disease. Ann N Y Acad Sci. (2010) 1207:E2–6. 10.1111/j.1749-6632.2010.05804.x 20961302

[50] Carlson KC Parnassus WN Klatt EC. Thoracic lymphangiomatosis. Archiv Pathol Lab Med. (1987) 111:475–77. 3105517

[51] Jones MP. Vascular diseases in birds of prey. J Exot Pet Med. (2013). 22:348–57. 10.1053/j.jepm.2013.10.012

[52] Bavelaar FJ Beynen AC. Atherosclerosis in parrots. A review. Vet Q. (2004) 26:50–60. 10.1080/01652176.2004.9695168 15230050

[53] Byers PH. Disorders of collagen biosynthesis and structure. In: Scriver CR Beaudet al Sly WS Valle D editors. The Metabolic and Molecular Bases of Inherited Disease. 7th ed. New York, NY: McGraw-Hill (1995). p. 4029–77.

[54] Mitchell RN Halushka MK. Blood vessels. In: Cotran RS Kumar V Robbin SL Schoen FJ editors. Pathologic Basis of Disease. Philadelphia, PA: ELSEVIER. (2020). p. 485–27.

[55] Nagata H Yonemura Y Canbay E Ishibashi H Narita M Mike M. Differentiating a large abdominal cystic lymphangioma from multicystic mesothelioma: report of a case. Sur Today. (2014) 44:1367–70. 10.1007/s00595-013-0654-x 23807639

[56] Rossi G Galosi L Berardi S Piano MA Robino P Rose T. Neck Kaposiform haemangioendothelioma in a Fischer's lovebird (*Agapornis fischeri*). Res Vet Sci. (2016) 106:112–5. 10.1016/j.rvsc.2016.03.018 27234547

[57] Wigle JT Harvey N Detmar M Lagutina I Grosveld G Gunn MD. An essential role for Prox1 in the induction of the lymphatic endothelial cell phenotype. EMBO J. (2002) 21:1505–13. 10.1093/emboj/21.7.1505 11927535 PMC125938

[58] Kato S Shimoda H Ji RC Miura M. Lymphangiogenesis and expression of specific molecules as lymphatic endothelial cell markers. Anat Sci Int. (2006) 81:71–83. 10.1111/j.1447-073X.2006.00142.x 16800291

[59] Álvarez-Hernán G Hernández-Núñez I Rico-Leo EM Marzal A de Mera-Rodríguez JA Rodríguez-León J. Retinal differentiation in an altricial bird species, *Taeniopygia guttata*: an immunohistochemical study. Exp Eye Res. (2020) 190:107869. 10.1016/j.exer.2019.107869 31705900

